# High Quality Coal Foreign Object Image Generation Method Based on StyleGAN-DSAD

**DOI:** 10.3390/s23010374

**Published:** 2022-12-29

**Authors:** Xiangang Cao, Hengyang Wei, Peng Wang, Chiyu Zhang, Shikai Huang, Hu Li

**Affiliations:** 1 School of Mechanical Engineering, Xi’an University of Science and Technology, Xi’an 710054, China; 2 Shaanxi Provincial Key Laboratory of Intelligent Testing of Mine Mechanical and Electrical Equipment, Xi’an 710054, China

**Keywords:** coal foreign object detection, data augmentation, GAN, self-attention, depthwise separable convolution

## Abstract

Research on coal foreign object detection based on deep learning is of great significance to safe, efficient, and green production of coal mines. However, the foreign object image dataset is scarce due to collection conditions, which brings an enormous challenge to coal foreign object detection. To achieve augmentation of foreign object datasets, a high-quality coal foreign object image generation method based on improved StyleGAN is proposed. Firstly, the dual self-attention module is introduced into the generator to strengthen the long-distance dependence of features between spatial and channel, refine the details of the generated images, accurately distinguish the front background information, and improve the quality of the generated images. Secondly, the depthwise separable convolution is introduced into the discriminator to solve the problem of low efficiency caused by the large number of parameters of multi-stage convolutional networks, to realize the lightweight model, and to accelerate the training speed. Experimental results show that the improved model has significant advantages over several classical GANS and original StyleGAN in terms of quality and diversity of the generated images, with an average improvement of 2.52 in IS and a decrease of 5.80 in FID for each category. As for the model complexity, the parameters and training time of the improved model are reduced to 44.6% and 58.8% of the original model without affecting the generated images quality. Finally, the results of applying different data augmentation methods to the foreign object detection task show that our image generation method is more effective than the traditional methods, and that, under the optimal conditions, it improves AP_box_ by 5.8% and AP_mask_ by 4.5%.

## 1. Introduction

Coal is the most abundant and widely distributed conventional energy source in the world, and is also an important strategic resource [1]. In the process of coal production, coal is frequently mixed with gangue, woods, anchor rods, woven bags, iron, and other foreign objects, which seriously affects the safe, efficient, and green production of coal mines [2,3], and it is urgent to choose an automated and intelligent foreign object detection and separation method [4]. With the rapid development of coal mine intelligence, foreign object detection methods based on deep learning have received wide attention from scholars [5]. However, deep learning is a data-driven method [6], and the training process usually requires the support of large datasets to prevent model overfitting. For example, Zhang [7] used 18,714 images to train a semantic segmentation model containing four types of coal foreign objects and achieved 91.24% segmentation accuracy. Hao [8] used 2300 images to train a large foreign object detection model, and the authors noted in their conclusion that the total number of foreign object samples had limited the detection accuracy. According to the investigation, there are significant differences in the features of foreign bodies between different coal mines, and there is no public high-quality foreign object dataset to support model training, due to constraints such as sparse foreign object sample content in regular production and harsh foreign object collection conditions. Therefore, achieving efficient data augmentation on limited datasets becomes the key to improve foreign object detection performance.

Image data augmentation methods are mainly divided into traditional augmentation and GAN (Generative Adversarial Network) based augmentation. Traditional augmentation methods are based on image processing, which mainly include geometric and color transformations, adding noise and filtering, random elimination, image blending, etc. [9,10]. At present, most of the research on coal foreign object image data augmentation focuses on traditional methods [11,12]. Traditional augmentation methods have great efficiency in image preprocessing, but they do not fundamentally solve the problem of insufficient foreign object data diversity, and are of limited help in foreign object detection. With the development of the data augmentation, GAN provides a new direction for image data augmentation, which can effectively solve the problems of small samples and data imbalance by generating a large number of samples with the same distribution as the real dataset [13]. The early GAN has disadvantages, such as gradient disappearance, gradient explosion, convergence difficulties, and low resolution of generated images. With scholars’ in-depth research on GAN, several works, such as WGAN [14], WGAN-GP [15], DCGAN [16], ProGAN [17], and BigGAN [18], have been introduced to gradually improve the training stability of GAN and the resolution of generated images, which laid the foundation for its application in machine vision tasks. Shi [19] added Wasserstein divergence to WGAN to improve the diversity of defect samples on the surface of micro-electromechanical systems (MEMS), and the experimental results showed that the mAP and F1 scores of defect detection were improved by 8.16% and 6.73%, respectively, after data augmentation. Deng [20] applied WGAN-GP to data augmentation for facial expression recognition, and improved the recognition accuracy of multi-angle facial expressions. In the field of coal mining, the application of GAN is still in the initial stage. In order to improve the accuracy of coal and rock recognition, Wang [21] introduced a pyramid structure in consinGAN to generate coal and rock images with a resolution of up to 250 × 250 by increasing from coarse to fine. Wang [22] incorporated a new convolution module in DCGAN to expand the resolution of generated images from 64 × 64 to 512 × 512, thus facilitating the training of coal-gangue detection models. The above research demonstrates the feasibility of applying GAN to visual recognition and detection tasks. However, coal foreign objects with diverse categories and irregular shapes impose higher requirements on the quality of the dataset, and the images generated by the above methods fail to achieve a balance between resolution and diversity to meet the needs of this paper.

StyleGAN [23] is one of the best performing generative frameworks, which proposes a combination of style module (AdaIN) and progressive generation strategy for generative networks that can generate 1024 × 1024 high-resolution images while increasing the diversity of datasets, and has been widely used in many fields [24,25]. Nevertheless, limited by the receptive field of the convolutional structure, it is difficult for the generative model to capture the long-term and global features of the object, which makes CNN-based models such as StyleGAN detect detail defects, such as teardrop artifacts and shape distortion, in the generated images. Aiming at such problems, Li [26] proposes a generative model based on the WD (wide and deep feature extraction block) module, which improves the quality of the generated images by combining the depth information extracted by ResNet with the global information extracted by Inception V1. The WD module contains a large number of convolution operations and has a large impact on the complexity of the model. SAGAN [27] introduced spatial self-attention into GAN to construct connections between different regions, and verified the ability of the self-attention mechanism to grasp the local details of the generated images; however, it ignored the relationship between channels of feature maps, which led to anomalous structures in the generated images. Yang [28] adds channel attention to SAGAN to further refine the texture, structure, and other features of generated images, but the suppressive effect of channel attention also leads to the absence of partial details.

In summary, devoting attention to the need for fast and massive acquisition of high-resolution, high-diversity, and sufficiently detailed coal foreign object images, to provide data support for coal foreign object detection models, a novel high-quality coal foreign object image generation method based on StyleGAN-DSAD is proposed. The main contributions of this paper are summarized as follows:

- We introduced a dual self-attention module (DSAM) into the generator of StyleGAN to strengthen the long-distance dependence of features between spatial and channel, which could refine the details of the generated images and solve the problems of artifacts, distortions, and front background adhesion in the generated images.
- Through research and experiments, we found that the discriminator part has little effect on the quality of the generated images; thus, we replaced the standard convolution in the discriminator with a depthwise separable convolution (DSC) to reduce the time and space complexity of StyleGAN and improve the training efficiency.
- Compared with the baseline method, images generated by the proposed method can generate better quality and more diverse foreign object images. Meanwhile, the accuracy of coal foreign object detection was effectively improved after data augmentation using the proposed method, indicating that the application of StyleGAN-DSAD to coal foreign object image augmentation is feasible.

## 2. Related Work

### 2.1. Generative Adversarial Network

**Figure 1:**
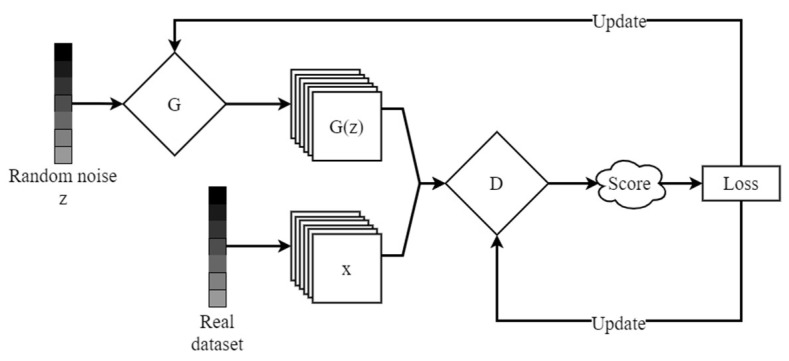
Framework of GAN.

The generative adversarial network is based on the idea of a zero-sum game, as shown in Figure 1, which mainly consists of a generator G and a discriminator D [13]. The input of G is a set of random noise z and the output is generated image, and the input of D is the image and the output is the probability that the input image is a real sample.

GAN performs model optimization by a maximum–minimum optimization objective function, as shown in Equation (1):

(1) $$\underset{G}{min}\underset{D}{max}V\left(D,\;G\right)=E_{x\sim P_{data}\left(x\right)}\left[log\left(D\left(x\right)\right)\right]+\;E_{z\sim P_{z}\left(z\right)}\left[log(1-D\left(G\left(z\right)\right)\right)]$$

where x and z denote the real data and input noise, respectively, and $P_{data}\left(x\right)$ and $P_{z}\left(x\right)$ denote the probability distributions obeyed by the real data and input noise, respectively.

During the entire training process, the generator and the discriminator trained alternately. The parameters of generator were frozen during the training of the discriminator, and vice versa during the training of the generator. As the training process is executed, the parameters of the generator and discriminator will reach an equilibrium (Nash equilibrium), at which time the generator could generate a large number of fake images that resemble real samples.

### 2.2. StyleGAN

**Figure 2:**
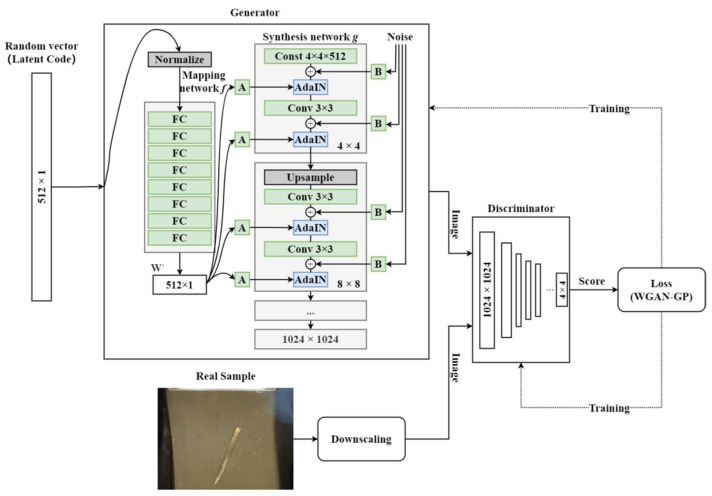
The network structure of StyleGAN.

StyleGAN is proposed by Karras to generate high resolution and high diversity images. As shown in Figure 2, it is mainly composed of a generator (consisting of a mapping network and a synthesis network), a discriminator, and a loss function.

- Mapping network

The role of the mapping network is to cooperate with the synthesis network to control the visual features of the generated images. The input of the mapping network is a set of Gaussian distributed random vectors Z, and the input vectors are encoded into intermediate vectors W’ of the same size with feature deconvolution through eight fully connected layers. The different elements in W’ are used to control the image generation style, such as color, texture, and shape.

- Synthesis network

**Figure 3:**
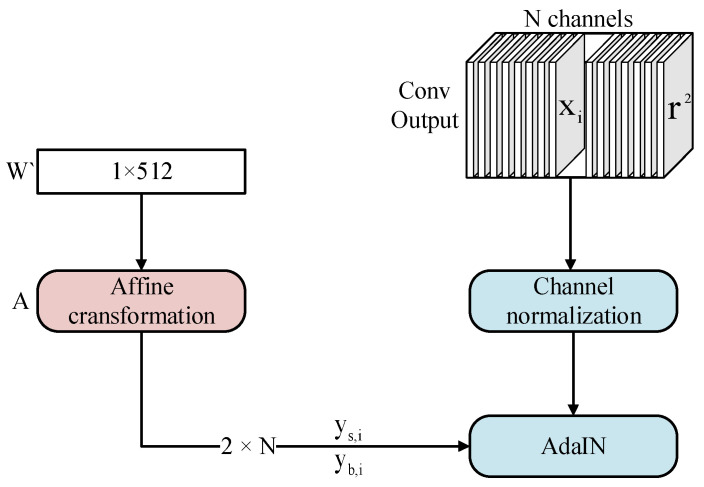
The implementation process of AdaIN.

The synthesis network consists of several sub-networks, for which the initial input is a constant feature of size 4 × 4 × 512, and after forward propagation, the resolution of the generated image increases smoothly from 4 × 4 to 8 × 8 to the highest resolution set, which solves the problems of large training costa and crashing of the training process in the direct generation method. The synthesis sub-network consists of an upsampling layer, a convolutional layer, an Adaptive Instance Normalization (AdaIN) module, a control vector A, and a noise B, which act jointly to enhance the diversity of the generated images. The noise is first added to the feature x along each channel, and then the AdaIN module influences the generation style, as shown in Figure 3 and Equation (2).

(2) $$AdaIN\left(x_{i},y\right)=y_{s,i}\frac{x_{i}-\mu \left(x_{i}\right)}{\sigma \left(x_{i}\right)}+y_{b,i}$$

where $x_{i}$ denotes the feature map of layer i, $\mu \left(x_{i}\right)$ and $\sigma \left(x_{i}\right)$ denote the mean and variance, respectively, and $y_{s,i}$ and $y_{b,i}$, denote the deflation factor and deviation factor, respectively.

Firstly, the mean and variance of the convolutional layer output are normalized by channel, while W’ is expanded into a deflation factor and deviation factor by a learnable affine transformation A. The two factors are then weighted and summed with the convolution layer to complete W’s influence on the original output. AdaIN is added after each convolution operation; it affects each sub-network twice with different styles affected by images at different resolutions.

- Discriminator

The discriminator is like an inverse of the generator, which reduces the input image to a feature map of the same size as the input through filtering, downsampling, and convolution. The feature map is scored by a fully connected layer to judge the input image quality.

- Loss function

The loss function consists of two parts: generator loss and discriminator loss. The generator uses a standard loss function. The discriminator loss uses WGAN-GP [15], which improves the stability of the model training process by combining the Wasserstein loss with the gradient penalty. The loss functions are as follows:

(3) $$L_{G}=-[E_{G\left(z\right)\sim p_{g}}\left[D\left(G\left(z\right)\right)\right]]$$

(4) $$L_{D}=E_{G\left(z\right)\sim p_{g}}\left[D\left(G\left(z\right)\right)\right]-E_{x\sim p_{r}}\left[D\left(x\right)\right]+\lambda E\left[{\left(\nabla_{\hat{x}}D{\left(\hat{x}\right)}_{2}-1\right)}^{2}\right]$$

where $p_{g}$ denotes the distribution of generated data and $p_{r}$ denotes the distribution of real data, $E_{G\left(z\right)\sim p_{g}}\left[D\left(G\left(z\right)\right)\right]$ denotes the mathematical expectation when the generated data are used as inputs to the discriminator, $E_{x\sim p_{r}}\left[D\left(x\right)\right]$ denotes the mathematical expectation when the real data are used as inputs to the discriminator, λ (=10) denotes the penalty coefficient of gradient penalty item, and $\hat{x}$ denotes the gradient penalty object sampled uniformly from the generated and real data, x=(1−ε)G(z), ε∈[0, 1].

## 3. Proposed Methods

Coal foreign object detection usually uses features such as shape, texture, and color of the foreign object as the main bases for detection, which places higher demand on the quality, detail, and diversity of the generated images. StyleGAN has a clear advantage in generating high-resolution images, but there are still the following problems when directly applying it to coal foreign object image generation:

- The limited receptive field of the convolutional structure makes it difficult to learn global, long-term dependencies between features, resulting in missing details in key parts of the generated foreign object images [27], producing the phenomena of artifacts, shape distortion, and front background adhesion.
- The multi-level convolutional structure leads to a large number of model parameters, which increases the time and space complexity of the model training process.

**Figure 4:**
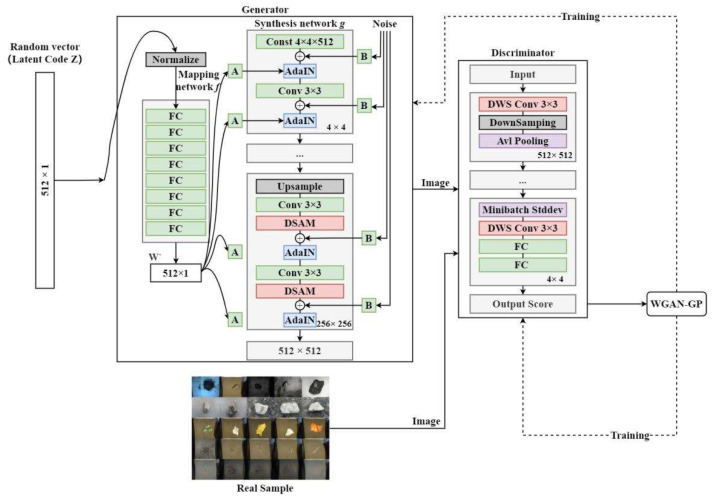
The network structure of StyleGAN-DSAD.

Thus, in order to improve the quality of generated images and assist in foreign object detection model training, this paper makes corresponding improvements to StyleGAN for the above problems, and the structure of the improved StyleGAN is shown in Figure 4. Firstly, to match the original dataset (resolution is 640 × 480), the maximum resolution of the generated images is adjusted to 512 × 512. Secondly, to solve the impact of receptive field limitation on the quality of the generated images, DSAM is introduced in the last three sub-networks of the synthesis network, which enables the synthesis network to capture the details of image shape and texture by learning the spatial interdependence of features and depict more detailed and realistic images, and to distinguish the foreground and background information more accurately by learning the channel interdependence of features and improve the phenomenon of front and background adhesion. Finally, to address the inefficiency caused by the large number of network parameters, we replaced the standard convolution in the discriminator with DSC, to reduce the number of network parameters and improve the training efficiency.

### 3.1. DSAM

**Figure 5:**
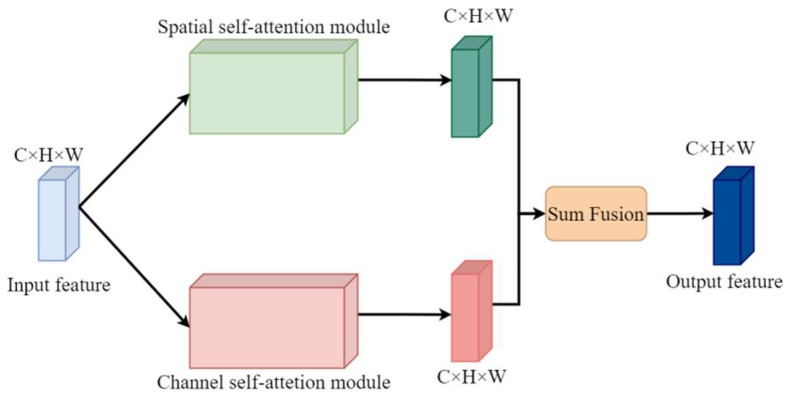
The principle diagram of DSAM.

It has beenshown that the introduction of the self-attention mechanism in the generative network helps to model the long-distance and multi-level dependencies in the generated images, and improves the generation quality. To solve the low quality problems such as artifacts, distortions, and adhesions in the foreign object images generated by StyleGAN, the DSAM [29] is introduced in the generator of StyleGAN, and its principle is shown in Figure 5.

The original feature map is fed into two modules of DSAM: Spatial Self-Attention Module and Channel Self-Attention Module. The refined feature maps of spatial and channel are obtained by extracting the interdependencies of features in spatial and channel and adding them with the original features in the two modules. Finally, the two output features are fused to obtain features with rich context information, thus improving the quality of the generated images. The entire process is as follows:

(5) $$A’=A_{S}\left(A\right)+A_{C}\left(A\right)$$

where A denotes the input feature map, and $A_{S}\left(*\right)$ and $A_{C}\left(*\right)$ denote the spatial and channel attention operations on the input features, respectively. The final refined feature map A’ is obtained by element-by-element summation of the two parts of the result.

**Figure 6:**
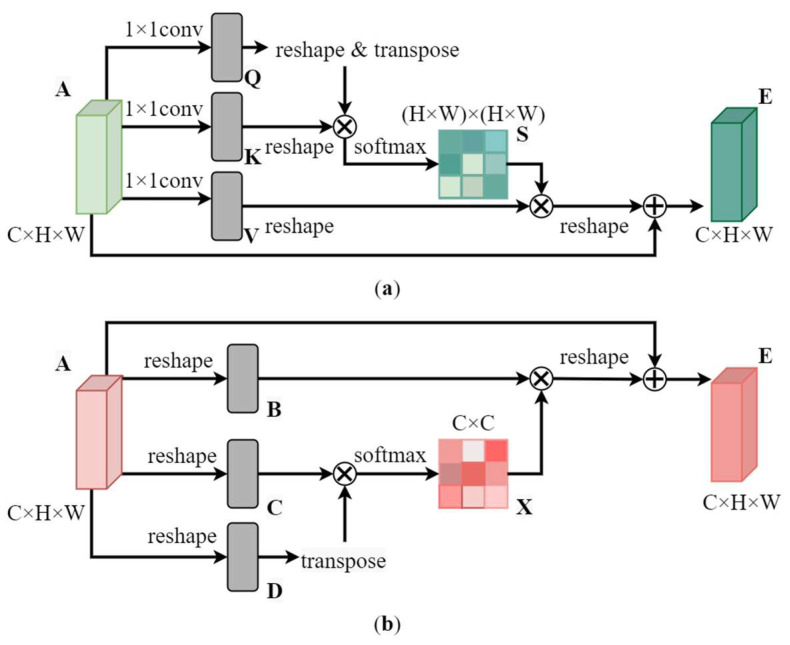
The details of spatial self-attention and channel self-attention. (**a**) Spatial self-attention module; (**b**) Channel self-attention module.

The specific details of the Spatial and Channel Self-Attention Modules are shown in Figure 6.

The Spatial Self-Attention Module is shown in Figure 6a. Firstly, the features map A∈R*^C×H×W^* is fed into three 1 × 1 convolution layers to obtain new feature maps *Q*, *K*, *V*(*Q*, *K*, *V*∈R*^C×H×W^*); subsequently, the shapes of *Q*, *K*, *V* are adjusted to *C × N* (*N = H × W*), the transpose of *Q* is matrix multiplied with *K* to obtain the spatial similarity measures of *Q* and *K*, and the multiplied results are processed by softmax to obtain the spatial attention map *S*∈R*^N×N^*. Finally, the feature *V* is multiplied with the attention matrix *S*, and the shape is adjusted back to *C × H × W*. The feature is then multiplied by a scale factor *α* and perform a element-wise sum operation with *A* to obtain the spatial refined feature map *E*. *S* and *E* are calculated as in Equation (6) and Equation (7), respectively.

(6) $$\;S_{ji}=\frac{exp\left(Q_{i}\cdot K_{j}\right)}{\sum_{i=1}^{N}exp\left(Q_{i}\cdot K_{j}\right)}$$

where $S_{ji}$ denotes the influence of the *i*-th position on the *j*-th position.

(7) $$E_{j}=\alpha \overset{N}{\underset{i=1}{\sum }}\left(S_{ji}V_{i}\right)+A_{j}$$

where *α* is initialized as 0 and gradually learns to allocate more weight, $A_{j}$ indicating the original feature map.

The Channel Self-Attention Module is shown in Figure 6b. Unlike the spatial attention module, the channel attention module computes the channel attention map directly from the original feature map *A*. The reason is that the original feature map better maintains the relationship between different channel feature maps. Firstly, the feature map *A* is adjusted to three features of shape R*^C×N^*(*N = H × W*), denoted as *B*, *C*, and *D*. Subsequently, the result of multiplying the transpose matrix of *D* with *C* is fed into the softmax layer to obtain the channel attention map *X*∈R*^C×C^*. Finally, the feature *B* is multiplied with the attention matrix *X* and the shape is adjusted back to *C × H × W*. The feature is then multiplied by a scale factor *β* and perform a element-wise sum operation with *A* to obtain the channel refined feature map *E. X* and *E* are calculated as in Equation (8) and Equation (9), respectively.

(8) $$\;X_{ji}=\frac{exp\left(C_{i}\cdot D_{j}\right)}{\sum_{i=1}^{C}exp\left(C_{i}\cdot D_{j}\right)}$$

where $X_{ji}$ denotes the influence of the *i*-th channel on the *j*-th channel.

(9) $$E_{j}=\beta \overset{C}{\underset{i=1}{\sum }}\left(X_{ji}B_{i}\right)+A_{j}$$

where *β* is initialized to 0 and gradually learns to allocate more weight, $A_{j}$ indicating the original feature map.

From Equation (5) to Equation (9), it is clear that DSAM is a “plug-and-play” module that does not affect the size and dimension of the input features, and can be seamlessly connected to all parts of the model. Meanwhile, it is tested that inserting the DSAM module to the deep sub-networks of the generator can achieve better generative results. Thus, DSAM is chosen to be inserted to the last three sub-networks of the generator. As shown in Figure 4, each sub-network of generator contains a convolution operation and a noise addition operation before AdaIN module, and our DSAM is inserted between the two operations. Firstly, the input size and dimension of the DSAM is adjusted according to the input features (the feature map size of the final three subnetworks is divided into $R^{128\times 64\times 64}\;$, $R^{64\times 128\times 128}$, and $R^{32\times 512\times 512}$), and then the refined features of the output of the DSAM are subsequently added with noise B and then fed into the AdaIN module, so that the model generates better quality and more diverse foreign object images.

### 3.2. DSC

**Figure 7:**
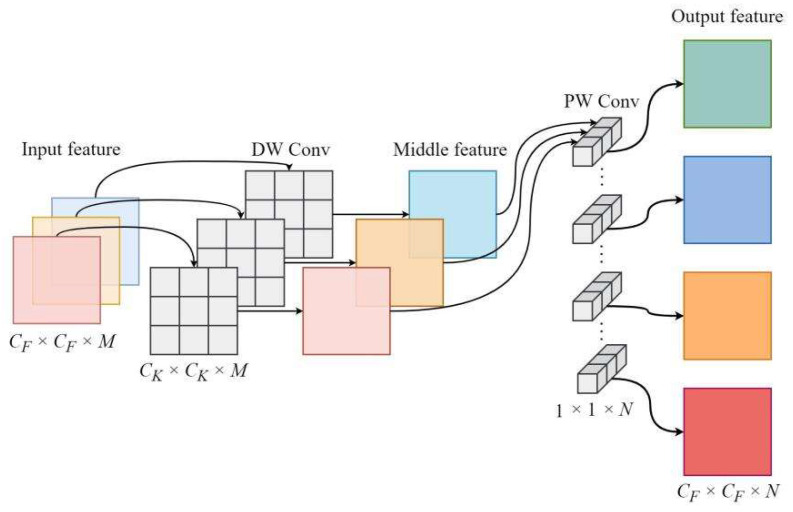
Depthwise separable convolution.

Depthwise separable convolution was first proposed in mobilenetV1 [30], which reduces the number of computational amounts and the number of parameters of CNN, at the cost of losing a small amount of accuracy, by decomposing the standard convolution into Depthwise (DW) Convolution and Pointwise (PW) Convolution. For GANs, the discriminator’s results for true and false samples only reflect the loss value of the current GAN model back propagation, which has little impact on the quality of the generated images [26]. Therefore, in this paper, we introduced the depthwise separable convolution in the StyleGAN’s discriminator to speed up the overall training efficiency of the network while reducing the complexity of network. The depthwise separable convolution structure is shown in Figure 7.

As can be seen from Figure 7, DSC is mainly divided into two parts: DW convolution and PW convolution. The DW convolution differs from the standard convolution in that each convolution kernel is responsible for only one channel, and its complexity reduction ratio is a multiple of the number of channels; the PW convolution is a standard convolution with a kernel size of 1 × 1, and its complexity reduction ratio is the product of the length and width of the convolution kernel. The linear combination of the two parts of convolution can meet the similar feature extraction effect of standard convolution and reduce the model computations and parameters to a certain extent. For example, suppose the input feature size is *C_F_ × C_F_ × M*, the output feature size is *C_F_ × C_F_ × N*, the convolution kernel size is *C_K_ × C_K_ × N*, and the computational amount and number of parameters of the standard convolution are:

(10) $$C_{SC}=C_{K}\times C_{K\;}\times M\times N\times C_{F\;}\times C_{F\;}$$

(11) $$P_{SC}=C_{K}\times C_{K\;}\times M\times N$$

where $C_{SC}$ and $P_{SC}$ denote the computational amount and number of parameters of the standard convolution, respectively.

The computational amount and number of parameters of DSC are:

(12) $$C_{DSC}=\left(C_{K}\times C_{K\;}\times M\times C_{F\;}\times C_{F\;}\right)+\left(M\times N\times C_{F\;}\times C_{F\;}\right)$$

(13) $$\;P_{DSC}=\left(C_{K}\times C_{K\;}\times M\right)+\left(M\times N\right)$$

where $C_{DSC}$ and $P_{DSC}$ denote the computational amount and number of parameters of the standard convolution, respectively, and the first half denotes DW convolution, the second half denotes PW convolution.

The complexity ratio of DSC to the standard convolution can be derived by combining Equation (10) to Equation (13):

(14) $$R_{C}=\frac{\left(C_{K}\times C_{K\;}\times M\times C_{F\;}\times C_{F\;}\right)+\left(M\times N\times C_{F\;}\times C_{F\;}\right)}{C_{K}\times C_{K\;}\times M\times N\times C_{F\;}\times C_{F\;}}=\frac{1}{N}+\frac{1}{C_{K}^{2}}\;$$

(15) $$R_{P}=\frac{\left(C_{K}\times C_{K\;}\times M\;\right)+\left(M\times N\;\right)}{C_{K}\times C_{K\;}\times M\times N\;}=\frac{1}{N}+\frac{1}{C_{K}^{2}}\;$$

As can be seen from Equations (14) and (15), the DSC has a larger compression in the computational amount and number of parameters compared to the standard convolution, and the efficiency of compression gets better as the complexity of the network increases. In this paper, we added the DSC in the discriminator part as shown in Figure 4. Except for the last sub-network, each DSC is placed before the downsampling operation for replacing the standard convolution, and in the last sub-network, the DSC is added before the fully connected layer (FC), and by the above way, the time and space complexity of the model could be reduced.

## 4. Experiments and Results

### 4.1. Dataset

**Figure 8:**
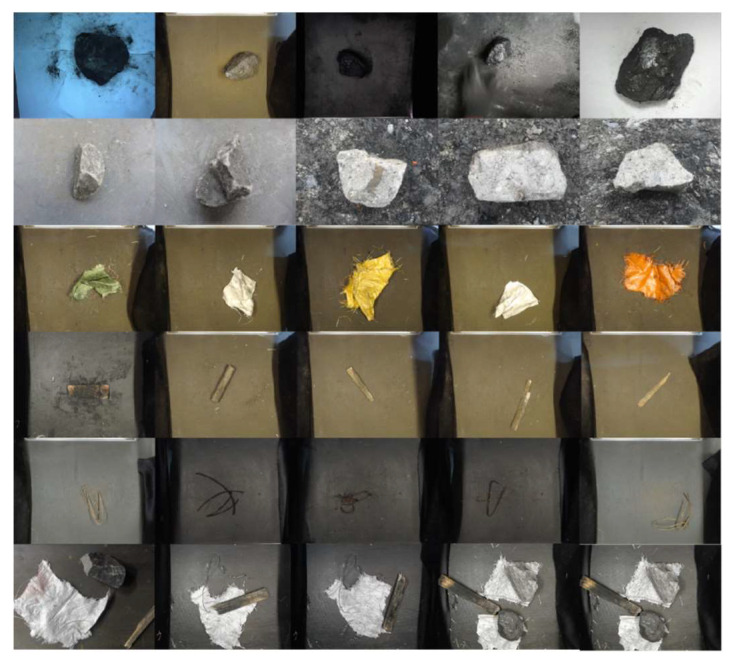
Images of coal foreign objects.

The training samples of the model were divided into two parts. The first part was collected in the field at the mine area, mainly including coal, gangue, and a small amount of other samples, and the second part samples were collected by scrap recycling, mainly including bags, wood, and iron. Our dataset consists of a total of 7825 images (including 1009 coal, 1050 gangue, 1495 wood, 1590 bags, 856 irons, and 1825 multi-target images), and the resolution is 640 × 480. Part of the foreign object image data as shown in Figure 8.

### 4.2. Experimental Settings

**Figure 9:**
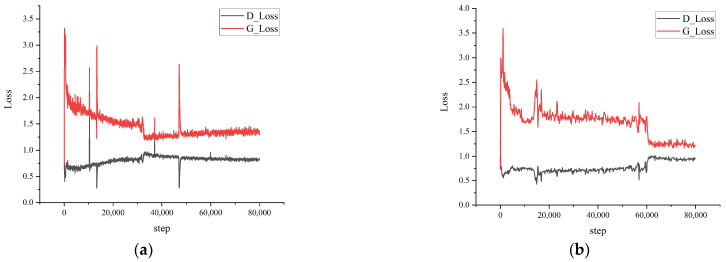

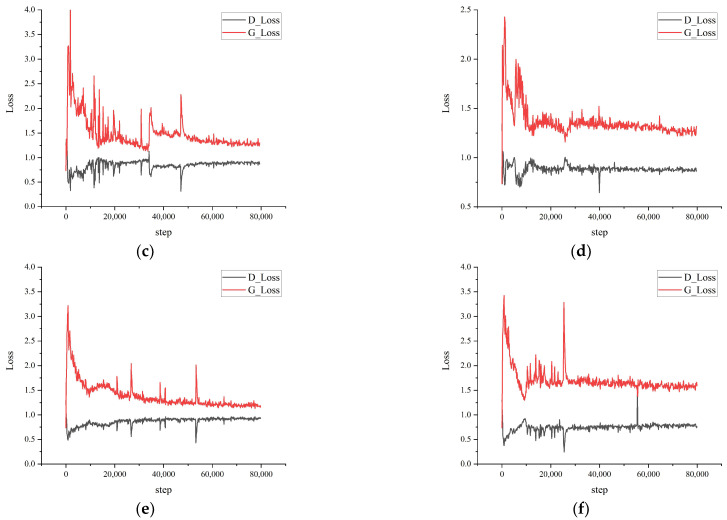
Loss curves of StyleGAN-DSAD. (**a**) all; (**b**) coal; (**c**) gangue; (**d**) bag; (**e**) iron; (**f**) wood.

Experiments were executed in Ubuntu 18.04 with the following configurations: the graphics card is an Nvidia Tesla A100 with 40 GB of video memory, the processor is an Intel Xeon 4212R with 128 GB of RAM, and the training environment is Python 3.9 + Pytorch 1.9.0 + Cuda 11.6. Adam optimizer was used to train the model with 80 K iterations and the batchsize is set to 24. The learning rate of the synthesis network and discriminator is set to 0.0005, and the learning rate of the mapping network is set to 0.005. Figure 9 shows the loss curve of our model; the loss curve fluctuates more in the early stage of training because the GAN needs to train two networks at the same time. As the training proceeds, the losses of the generator and discriminator gradually approach each other and level off at a certain number of iteration steps.

Experiments consisted of two major parts. The first part was an intuitive evaluation of the performance of StyleGAN-DSAD. Firstly, the quality and diversity of the generated images were evaluated, and the evaluation metrics were selected as the most credible Inception Score [31] (IS) and Frechet Inception Distance [32] (FID) in the field of GAN, where the IS is proportional to the quality and diversity of the generated images and the FID is inversely proportional to the quality and diversity of the generated images. Secondly, the model complexity is evaluated in terms of the model parameters (Params) and the total model training time (time/min). The second part tests the practical effect of data augmentation on the foreign object detection task, and the test model is chosen as the instance segmentation model BlendMask [33], and the average segmentation accuracy AP_mask_ and the average detection accuracy AP_box_ are used to evaluate the instance segmentation results.

### 4.3. Performance Evaluation of StyleGAN-DSAD

To verify the performance of the improved model in the image data augmentation task, GAN models are trained for five types of foreign objects and multi-target foreign objects, respectively. Subsequently, the quality, diversity, and model complexity of the images generated by each model were evaluated comprehensively. The comparison models were selected as DCGAN, ProGAN, BigGAN, StyleGAN, StyleGAN-DSAM (StyleGAN + Dual self-attention module), StyleGAN-DSC (StyleGAN + Depthwise separable convolution), and our StyleGAN-DSAD (StyleGAN + DSAM + DSC).

- Comparison of model generation quality and diversity

**Figure 10:**
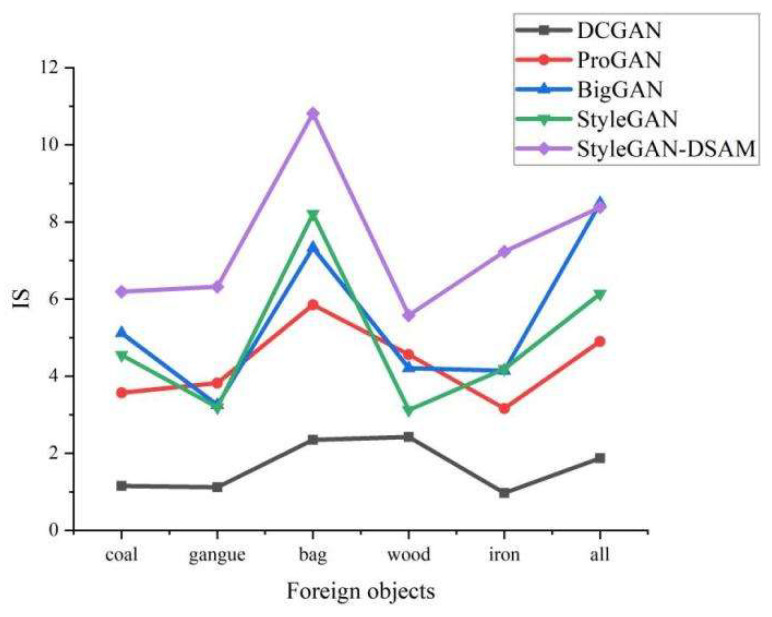
Comparison of IS.

**Figure 11:**
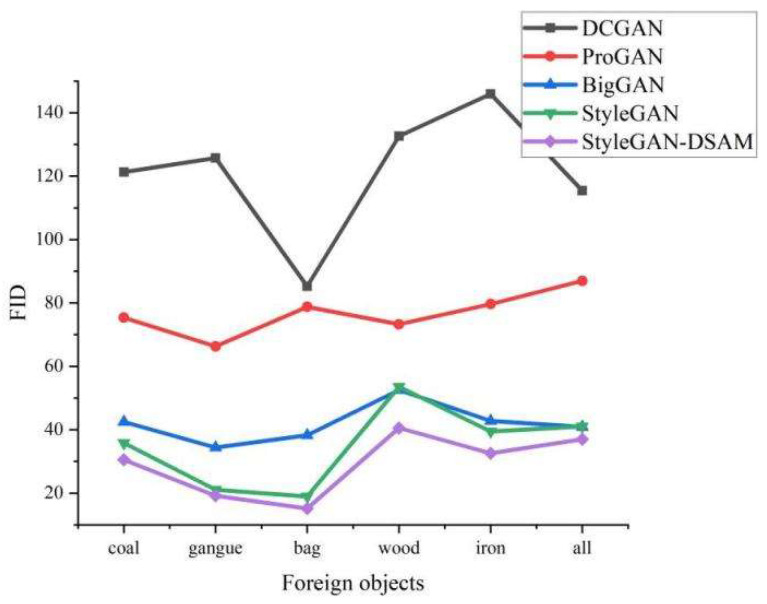
Comparison of FID.

The quality and diversity comparison results of different models are shown in Figure 10 and Figure 11. It can be seen from the figures that the FID and IS of DCGAN and ProGAN are far behind other networks and are not suitable for foreign object image generation. BigGAN is slightly better than StyleGAN in IS metrics, but its FID metrics relative to StyleGAN in coal, gangue, woven bag, and three types of foreign objects on the gap is large, indicating that BigGAN has certain advantages in generating high quality images, but, in contrast, StyleGAN can achieve a better balance between image quality and diversity.

- Comparison of model complexity

**Figure 12:**
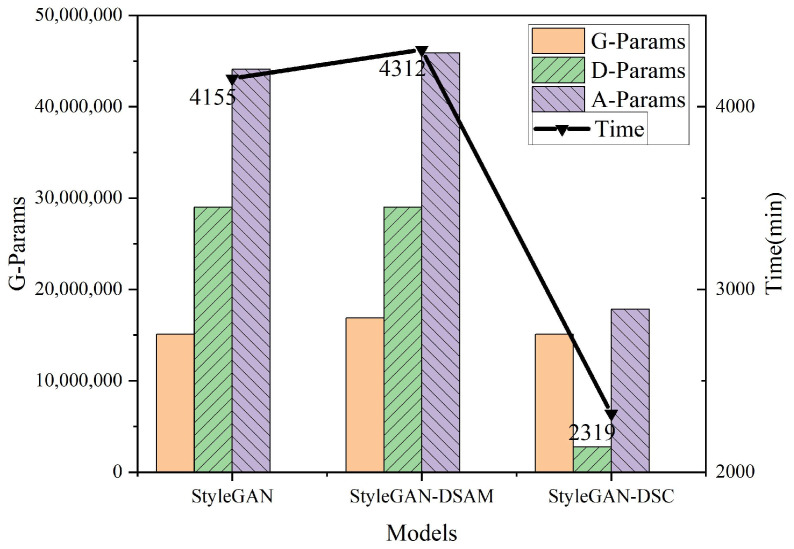
Comparison of model complexity.

The comparison results of the complexity of the StyleGAN model before and after optimization are shown in Figure 12. G-Params, D-Params, and A-Param indicate the parameters of the generator, discriminator, and whole model (sum of G-Params and D-Params), respectively, and Time indicates training time. As can be seen from the figure, the number of parameters accounted for by the discriminator part of StyleGAN is larger, about 65.8% of the whole network, so it is necessary to reduce the parameters of the discriminator. After the introduction of DSAM in the generator, the parameters of the generator rose a certain amount, accounting for about 11.8% of the original generator, and the total training time of the model increased by 157 min, accounting for 4% of the original training time; after replacing the standard convolution with a depthwise separable convolution, the number of parameters of the discriminator was compressed substantially, and the parameters were reduced to 9.5% of the original discriminator, the total parameters of the model were reduced to 40.5% of the original model, and the total training time is reduced to 57.7% of the original model. In summary, after the introduction of depthwise separable convolution, the number of parameters and training time of the model are effectively reduced, and the time and space complexity of the model is simplified.

- Overall performance evaluation

Table 1 shows the comparison results of the overall performance of the model after adding DSAM and DSC at the same time (IS and FID in the table indicate the average metrics of the generative models for each category, respectively). From the third row of Table 1, after adding only the DSAM module, the IS and FID metrics of model improve significantly, while the number of parameters and training time only increase by about 4% and 3.8%, respectively, which is due to the fact that the DSAM is added at the last three layers of the generative model, which only requires a small complexity cost. From the fourth row of Table 1, the IS and FID metrics each fluctuate slightly after adding the DSC module, indicating that the discriminator is not the main factor affecting the quality of the generated images, and the compression of its convolutional structure can substantially improve the model training efficiency and reduce the space occupation rate without changing the generation quality. From the fifth row of Table 1, after adding DSAM and DSC modules simultaneously, the quality and diversity of the StyleGAN-DSAD model generation is almost the same as that of StyleGAN-DSAM, but the number of parameters is reduced to 44.5% of the original model, the training time is reduced to 58.8%, and the comprehensive performance of the model is optimized.

- Comparison of actual generation effect

**Figure 13:**
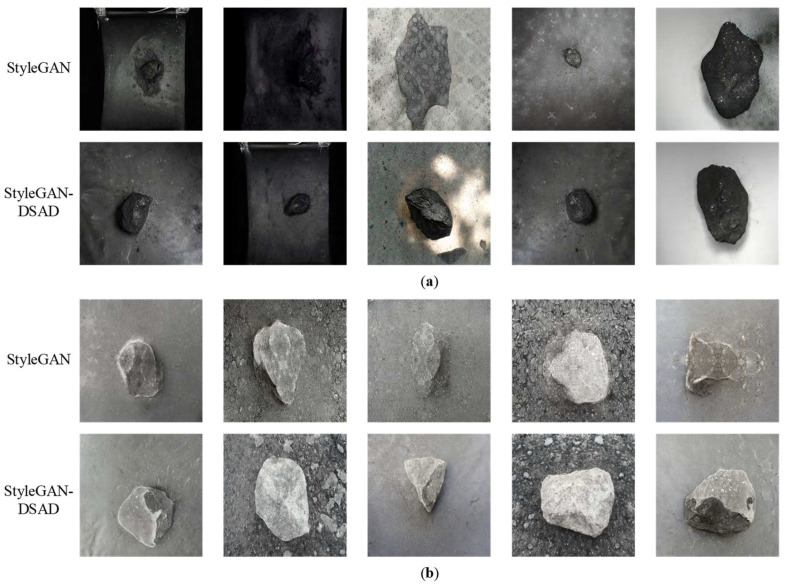

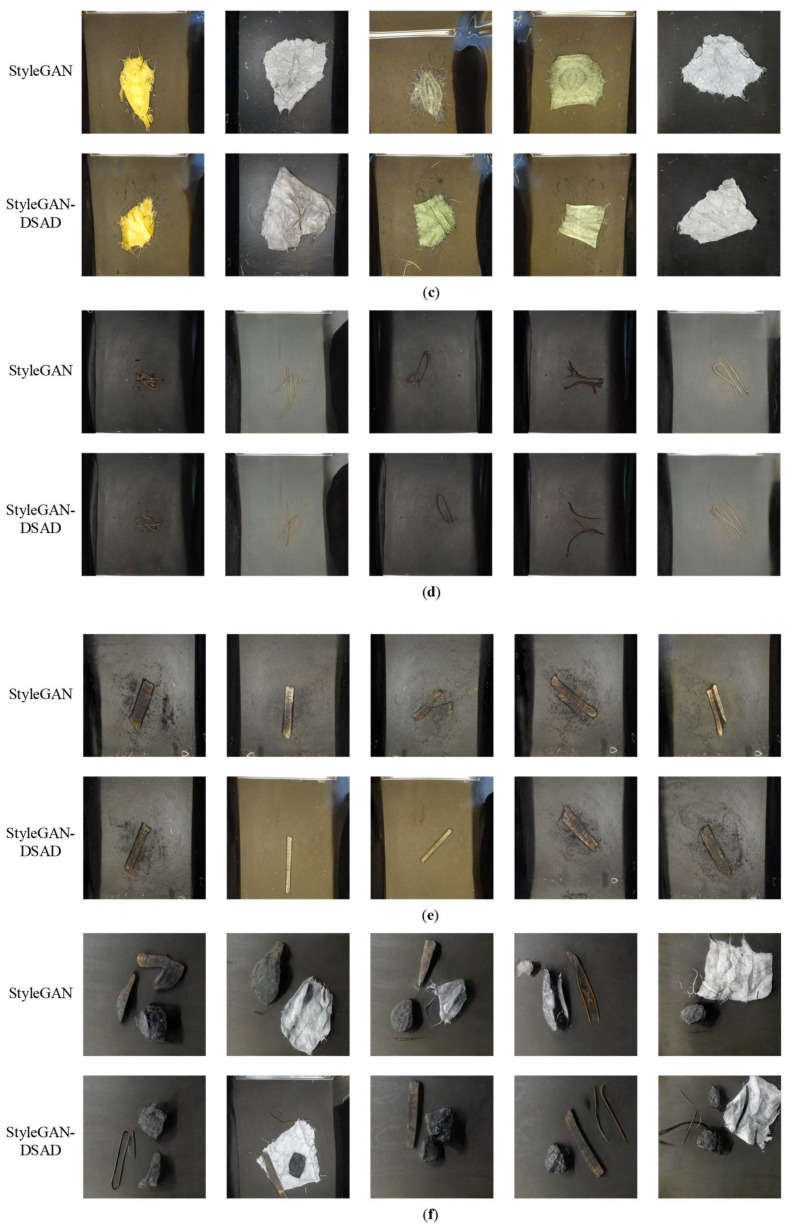
Comparison results of generated images. (**a**) coal; (**b**) gangue; (**c**) bag; (**d**) iron; (**e**) wood; (**f**) all.

In order to visually demonstrate the effect of the improved model, six types of foreign object images generated by StyleGAN and StyleGAN-DSAD were selected for comparison. As shown in Figure 13, the first row of each type of generated images was generated by StyleGAN, and the second row was generated by StyleGAN-DSAD. Observing the generated images of each group, it is obvious that the StyleGAN generated images have teardrop artifacts, and there are phenomena such as adhesion of the front background and shape distortion. For example, in column 2 of Figure 13a, the coal generated by StyleGAN seriously overlaps with the background, and in column 3, the shape and color of the StyleGAN generated images have produced more obvious distortion, and the generated images are no longer recognizable as coal; similar problems are shown in columns 3, 4, and 5 of Figure 13b, columns 3 and 4 of Figure 13c, columns 3, 4, and 5 of Figure 13d, column 4 of Figure 13e,f. On the contrary, observing StyleGAN-DSAD, the improvement of the generator makes the texture, contour, and color of the generated images clearer and more fitting to the original dataset. At the same time, problems such as front background adhesion and shape distortion are also well improved.

### 4.4. Practical Effects of Data Augmentation for Foreign Object Detection

To further demonstrate the effectiveness of our research, the instance segmentation framework BlendMask was selected as the foreign object detection model to test the actual effect of data augmentation. The original dataset is the same as that of the training StyleGAN used, and 20% and 10% are randomly selected as the validation set and test set. By replacing different training sets for experimental comparison, in order to balance the dataset, the final ratio of all kinds of foreign object data amplified is kept as 1.2:1.2:1:1:1.2:1 for coal, gangue, bag, iron and wood. The hardware environment for BlendMask training remains unchanged, and the deep learning environment is the dectectron2 framework under Python 3.6 + Pytorch 1.7.0. The training details are as follows: initialize the backbone weights and accelerate the model convergence using the ResNet101 pre-trained model on ImageNet. SGD is used to train the model with 48 K iterations and a batchsize of 8. The learning rate is changed using a warm up strategy, with the initial learning rate set to 0.002, and the learning rate is reduced by a factor of 10 at 60% and 90% of the total number of iterations. The remaining hyperparameters are kept the same as the original text.

Firstly, the usefulness of our method for the coal foreign object detection is verified by comparing the training results of the generated images with the real images, and the experimental results are shown in Table 2. As can be seen from the table, although there is a certain gap compared with the training results of real data, using the generated data as the training set can still improve the foreign object detection accuracy, and the training effect of the generated data is gradually improved with the increase in the training data. When the generated data is expanded to 10,000, the model accuracy is no longer improved, and the AP_box_ is 71.9% and the AP_mask_ is 62.6%, which are 3.9% and 4.9% different from the optimal results of the real data, indicating that although the generated images can support the training of foreign object detection model to a certain extent, the training of the generated model is generally a process of learning the distribution of the real dataset, and cannot completely replace the real data.

**Figure 14:**
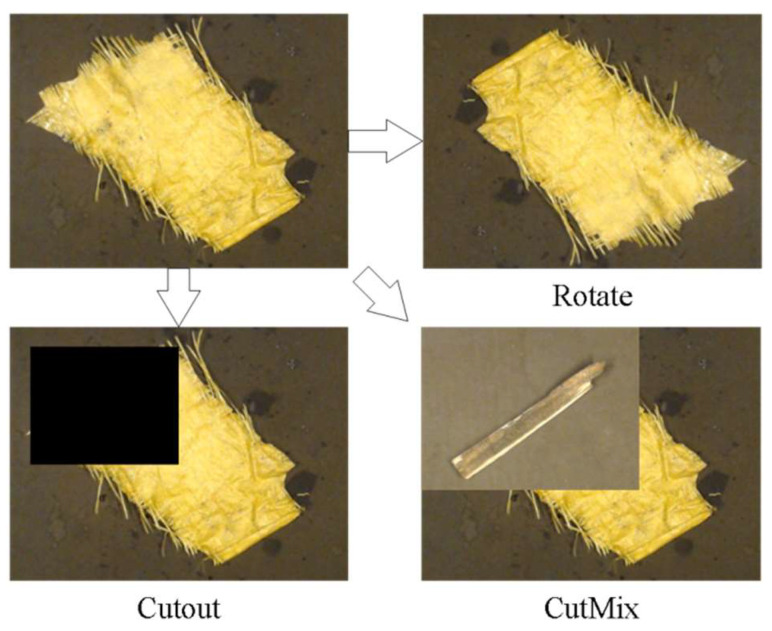
Traditional augmentation method.

Secondly, in order to verify the advantages and disadvantages of the generation method of this paper and the traditional data augmentation methods, comparison experiments were conducted under different augmentation methods, and the traditional data augmentation methods used are shown in Figure 14, which mainly include geometric transformation, Cutout, CutMix, and so on.

**Figure 15:**
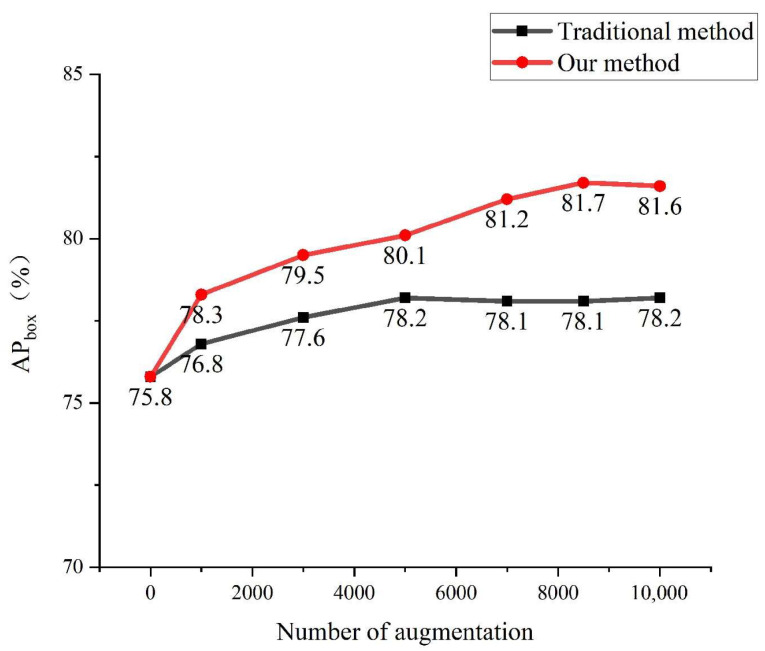
Comparison of AP_box_.

**Figure 16:**
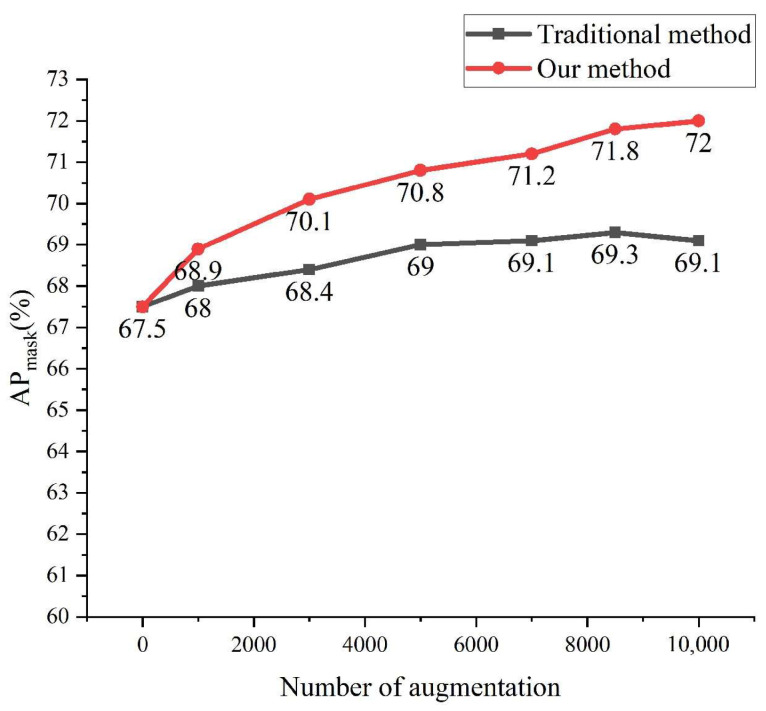
Comparison of AP_mask_.

The comparison results are shown in Figure 15 and Figure 16, where the horizontal coordinates indicate the original dataset plus different amounts of augmentation data. It can be seen that the performance of the foreign object detection model is improved by using both augmentation methods, but the generation method is more effective, because the images obtained by the traditional augmentation method are based on the original image, and the diversity is not enough, while the generation method improves the diversity of the dataset to a certain extent, thus better facilitating the model training. When the traditional data augmentation method augments the data to be basically the same as the original dataset, the model accuracy is no longer improved, and the final AP_box_ is improved by 2.4% and AP_mask_ is improved by 1.6%. When the data is expanded to 8000 in the generation method, the model accuracy is basically no longer improved, and when it is expanded to 10,000, the model performance reaches the optimum, and then the AP_box_ is improved by 5.8% and AP_mask_ is improved by 4.5%.

**Figure 17:**
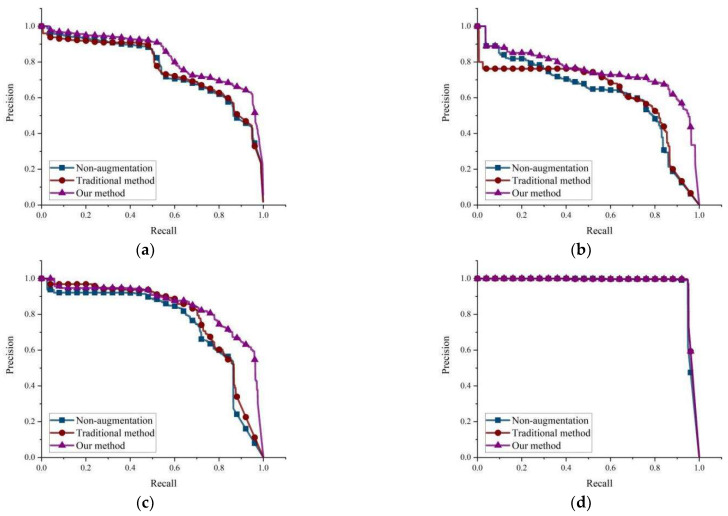

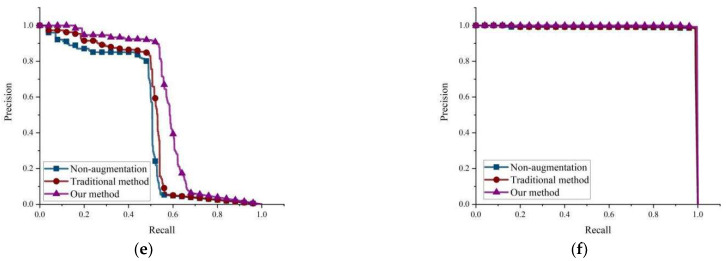
Comparison of PR curves. (**a**) all; (**b**) coal; (**c**) gangue; (**d**) bag; (**e**) iron; (**f**) wood.

The PR curves of each type of foreign object under different data augmentation methods are shown in Figure 17. From Figure 17d,f, it can be seen that the accuracy of the three datasets is close to each other because the characteristics of bag and wood are more obvious, and the accuracy of our method is slightly higher. From Figure 17a–c,e, it can be seen that the overall accuracy of the model and the detection accuracy of coal, gangue, and iron are significantly improved after using our method compared with non-augmentation and traditional augmentation methods, which indicates that our method has a better promotion effect on the detection of each type of foreign object.

**Figure 18:**
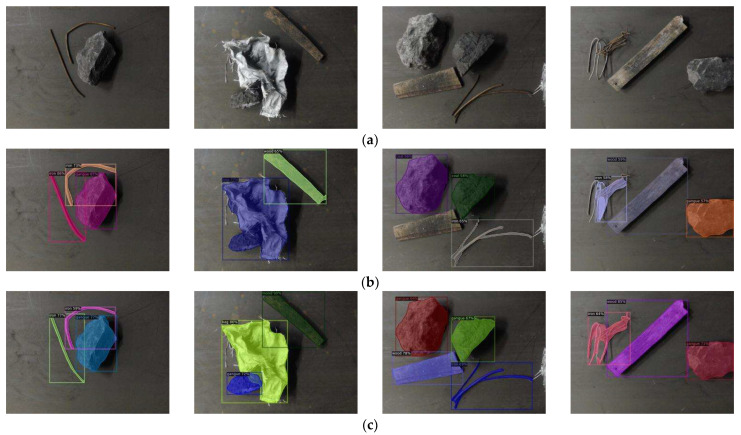
Comparison of detection effects before and after data augmentation. (**a**) Samples; (**b**) Before augmentation; (**c**) After augmentation.

The actual detection results of the model before and after data augmentation using the method in this paper are shown in Figure 18. It can be found that after the data augmentation, the foreign object detection effect is significantly improved, and the segmentation contour is clearer while effectively reducing the phenomenon of false detection and missed detection.

## 5. Conclusions

Our goal was to solve the problems of difficult training and low detection accuracy of coal foreign object detection models caused by the lack of datasets. In this paper, we perform foreign object data augmentation by image generation in order to improve the quality and diversity of foreign object datasets and facilitate foreign object detection model training. Specifically, a high-quality foreign object image generation method based on StyleGAN-DSAD is proposed. Firstly, the quality and diversity of the generated images are improved by introducing a dual self-attention module to improve the artifacts, shape distortion, and front background adhesion of the generated images; secondly, the number of parameters of the model is greatly reduced and the training efficiency of the model is improved by replacing the convolutional structure of the discriminator with a depthwise separable convolution; finally, a high-quality foreign object image with a resolution of 512 × 512 is generated to achieve coal foreign object data set expansion. The experimental results show that the improved model effectively improves the quality and diversity of the generated images, and the complexity of the model is greatly reduced. After data augmentation, the performance of the foreign object detection model is significantly improved compared with both non-augmentation and traditional augmentation methods, which effectively reduces the occurrence of false detection and missed detection, and proves the feasibility of applying StyleGAN-DSAD to the field of coal foreign object detection.

## Figures and Tables

**Table 1:** Overall performance evaluation of the models.

Model	IS	FID	A-Params	Params Rate/%	Time/min	Time Rate/%
StyleGAN	4.9	34.99	44,105,664	/	4155	/
StyleGAN-DSAM	7.42	29.1	45,884,736	104.0	4312	103.8
StyleGAN-DSC	4.57	34.8	17,849,340	38.9	2396	57.6
StyleGAN-DSAD	7.41	29.3	19,628,412	44.5	2443	58.8

**Table 2:** Comparison of training effect between generated image and real image.

Training Set	AP_box_/%	AP_mask_/%
1000 generated images	52.3	41.6
1000 real images	59.9	50.7
3000 generated images	60.0	51.5
3000 real images	68.0	59.6
5000 generated images	67.8	59.5
5477 real images	75.8	67.5
7000 generated images	70.0	61.6
8500 generated images	71.8	61.4
10,000 generated images	71.9	62.6

## Data Availability

The data presented in this study are available on request from the corresponding author. The data are not publicly available due to project confidentiality.
